# Morphofunctional changes following adenotonsillectomy of obstructive sleep apnea children: a case series analysis

**DOI:** 10.1186/s40510-022-00422-7

**Published:** 2022-08-08

**Authors:** Mariana M. Tinano, Helena M. G. Becker, Letícia P. Franco, Claudia P. G. dos Anjos, Vinícius M. Ramos, Carolina M. F. F. Nader, Joana Godinho, Henrique de Párcia Gontijo, Bernardo Q. Souki

**Affiliations:** 1 Outpatient Clinic for the Mouth-Breathers, Federal University of Minas Gerais, Av. Prof Alfredo Balena 190, Hospital São Geraldo, Belo Horizonte, MG 30.130-100 Brazil; 2 School of Dentistry, Orthodontics, University of Lisbon, Rua Professora Teresa Ambrósio, Cidade Universitária, 1600-277 Lisbon, Portugal; 3 Graduate Program in Dentistry, Orthodontics, Pontifical Catholic University of Minas Gerais, Rua Dom José Gaspar, 500 prédio 45, Belo Horizonte, MG 30535-000 Brazil

**Keywords:** Obstructive sleep apnea, Mouth breathing, Systolic pressure, Airway resistance, Adenoidectomy, Maxillofacial abnormalities

## Abstract

**Objective:**

To perform a case series analysis of the changes in the pulmonary artery systolic pressure (PASP), nasal inspiratory flow (NIF), upper airway volume, obstructive apnea/hypopnea index (OAHI), and the maxillomandibular three-dimensional (3D) morphology after adenotonsillectomy (T&A) of obstructive sleep apnea children (OSA).

**Materials and methods:**

Retrospective assessment of files from 1002 children screened between 2012 and 2020 in a hospital-based mouth-breather referral center. From this universe, 15 obstructive sleep apnea children (7 females; 8 males), ages 4.1 to 8.9 years old (mean age of 5.4 years ± 1.3), who presented indications of tonsillectomy and/or adenoidectomy were selected. The complete baseline examination (T0) was carried out before T&A and a second complete examination (T1) was made 18.7-month follow-up after T&A (ranging from 12 to 30 months). Eleven patients were submitted to T&A, and four patients had indications but did not receive authorization for surgery from the public health system. According to the protocol of the outpatient clinic for OSA patients, Doppler echocardiography, polysomnography, rhinomanometry, and computed tomography imaging was performed at (T0) and (T1).

**Results:**

PASP decreased 16.6% after T&A. NIF increased more in T&A children (40.3%) than in non-T&A children (16.8%). The upper airway volume increased in T&A and non-T&A children, but greater volumetric gain (45.6%) was found in the nasopharynx of T&A patients. OAHI did not change in six T&A children (55%) and three non-T&A children (75%). The maxilla displaced downward and backward relative to the cranial base in six T&A children (55%) and two untreated children (50%). Nine of the T&A children (85%) and three untreated children (75%) presented extensive condylar growth and increased mandibular length. The qualitative 3D assessment showed similar morphological 3D changes in T&A and non-T&A patients.

**Conclusion:**

Pulmonary artery systolic pressure decreased, nasal inspiratory flow increased, and nasopharynx volume increased following adenotonsillectomy, but obstructive apnea/hypopnea index and maxillomandibular morphology were similar in surgical and non-surgical patients.

## Introduction

The partial or total blockage of the airflow through the nasal cavity, nasopharynx, or oropharynx might lead patients to sleep disorder problems, including obstructive sleep apnea (OSA). OSA syndrome is currently one of the most critical concerns to healthcare providers worldwide because of adverse effects on the quality of life and the potential association with pulmonary artery hypertension [1, 2]. Even minor increases (less than 35 mm HG) of the pulmonary artery systolic pressure (PASP) might have deleterious consequences. The vasoconstriction may cause vascular remodeling and right ventricular overload [2, 3]. Orthodontic problems might also be caused by mouth breathing of OSA pediatric patients, as the maxillomandibular abnormal morphological changes [4, 5].

Polysomnography is the gold-standard record for OSA, and the derived Obstructive Apnea and Hypopnea Index (OAHI) is one of its objective parameters to quantify the severity of the obstruction. The OAHI index measures the number of episodes of apnea and hypopnea per hour of sleep, which is directly related to OSAS. Oximetry is one of the parameters in polysomnography. Oxyhemoglobin desaturation, measured by the mean minimum saturation (%SpO2), is predictive of the severity of sleep-disordered breathing and plays a significant role in the neural repercussions associated with OSAS. Rhinomanometry is the standard test for evaluating impairment of nasal inspiratory flow (NIF) and consists of the assessment of active breathing through one nasal cavity while the transnasal pressure difference is assessed in the other cavity. It is a dynamic exam that calculates flow, pressure, and nasal resistance. The percentage of nasal obstruction is calculated by the ratio between total nasal inspiratory flow and the expected total nasal inspiratory flow according to height and given in predictive values. Moreover, a positive association between upper airway obstruction and clinically maxillomandibular morphological changes has been previously reported [6–8]. The most common cause of OSA in children is adenotonsillar hypertrophy; therefore, adenotonsillectomy (T&A) is the most frequent therapeutic approach for OSA. Previous studies have reported an improvement in the polysomnographic parameters, clinical symptoms, and quality of life after adenotonsillectomy due to removing the obstructive factor and increasing the airway space and improvement in the nasal patency [9, 10].

Several studies have reported the impact of normalization of the mode of breathing following T&A on OAHI [11], NIF [11, 12], PASP [11–13], and maxillomandibular growth changes, [4–15] expectations might not meet reality in a clinical individual patient scenario. Recent publications reported no significant differences in dentofacial morphology after the surgical normalization of the mode of breathing, suggesting divergences in this topic [16–19].

The current knowledge on the skeletal morphology of mouth breathers is based on bi-dimensional (2D) imaging with all limitations of this method, such as magnification, distortion, and superimposition of anatomic structures [20]. Three-dimensional (3D) imaging generated by computed tomography (CT) opened a new horizon to health care providers [21–23].

Therefore, the purpose of the current study was to perform a case series analysis of the changes in the pulmonary artery systolic pressure (PASP), nasal inspiratory flow (NIF), upper airway volume, obstructive apnea and hypopnea index (OAHI), and of the maxillomandibular 3D morphology following adenotonsillectomy (T&A) of obstructive sleep apnea (OSA) children.

## Materials and methods

### Ethical approval

This case-series study was written according to the PROCESS guidelines to improve the quality of scientific reports [24]. It was based on the consultation of patients’ records from the Clinics Hospital of the University outpatient clinic for mouth breathers and academic hospital-based services for children with mouth breathing disorders. Mouth-breathing children ranging from 0 to 12 years old are accepted at this service. As this is a retrospective case series report, a priori sample size calculation was not performed.

The study was approved by the Institutional Ethics Committee (CAAE 43321914.2.0000.5149) and was registered at National Clinical Trials (RBR-3sq5hv). All patients and their parents signed informed consent before treatment. The institution is an academic referral center for patients with mouth breathing in a state with 21 million citizens. It is a federal institution run with public governmental funds. One thousand and two patients referred by pediatricians due to mouth breathing problems were screened and treated by a multidisciplinary team between 2012 and 2020. From this vast universe, it was found 15 obstructive sleep apnea children (7 females; 8 males), ages 4.1 to 8.9 years old (mean age of 5.4 years ± 1.3), who presented indications of tonsillectomy and/or adenoidectomy, and attended the inclusion and exclusion criteria described below.

### Inclusion criteria

The inclusion criteria were: (a) children under ten years old; (b) OSA diagnosed with polysomnography; (c) Brodsky’s grades 3 or 4 for palatine tonsils obstruction [25]; (d) adenoid hypertrophy greater than 75% diagnosed with flexible nasopharyngolaryngoscopy [26]; (e) indication of T&A; and (f) pre-treatment and follow-up records of CT scans, polysomnography, Doppler echocardiography, and rhinomanometry.

### Exclusion criteria

The exclusion criteria included: (a) genetic syndromes and neuromuscular disease; (b) perforation of the nasal septum; (c) pulmonary hypertension due to heart disease; (d) the previous history of adenotonsillectomy; (e) corticosteroids, nasal decongestants or antihistamines; (f) previous orthodontic treatment; (g) presence of any craniofacial disorder; and (h) presence of central apnea.

### Intervention

Eleven patients had been submitted to T&A. Four patients with the indication for the surgical approach of the impaired airways did not receive T&A because authorization from the municipality public health system was not given due to financial problems.

The complete baseline examination (T0) was carried out following the clinical screening consultation with the otorhinolaryngologist. T&A was performed within three months after the first consultation. A second complete examination (T1) was made 18.7 months follow-up after T&A (ranging from 12 to 30 months) in surgical individuals. In non-surgical individuals, T1 exams were collected following the governmental authorization for the surgical intervention, 18 to 24 months post T0. According to the protocol of the outpatient clinic for OSA patients, Doppler echocardiography, polysomnography, rhinomanometry, and computed tomography imaging were performed at (T0) and (T1).

### Methods

The Doppler echocardiography with color flow mapping was performed with a Philips IE33 device (Philips Healthcare, Amsterdam, Holland). The same pediatric echocardiographer, unaware of the patient’s medical history, performed the examination. The PASP was calculated by using the Bernoulli formula. The upper limit of PASP was considered to be 30 mmHg (Guidelines of the American Heart Association and American Thoracic Society, 2015).

The polysomnography was done with a computerized infant sleep recorder (Alice Recording System 5, Respironics, GA, USA) according to the protocol recommended by the American Academy of Sleep Medicine (AAMS 2014) [27]. An experienced sleep doctor staged the records. Pediatric OSAS is classified as normal (OAHI < 1), mild (OAHI between 1 and 5), moderate (OAHI between 5 and 10), and severe (OAHI ≥ 10), according to the International Classification of Disorders CIDS-3 (2014). The criterion adopted in the current investigation to define obstructive sleep apnea was OAHI > 1 event/hour. The mean minimum saturation (%SpO2) was calculated.

Rhinomanometry was performed with SRE 2000 N 010000300189 RHINOSCAN 0272CFB2 from RHINOSTREAM 03CC5C3. A single senior otorhinolaryngologist conducted the exams. The nasal inspiratory flow values were measured in the left nostril at a transnasal pressure of 150 Pa. The same radiology technician performed multislice computed tomography scanning (MSTC) using *Scanner multislice* 128 (Somatom, Siemens, Erlangen, Germany), with a tube tension of 120 kV, 240 mA, and 1.57 seconds of exposure time. The patients were laid down in the supine position during the scan process, which better represents the position of the patients during sleep [28].

### CT’s image analysis

Using the CT scans, the volumetric measurements of the nasal cavity, nasopharynx, and oropharynx were taken by the same orthodontist, using 11.7 Dolphin Imaging software (Dolphin Imaging & Management Solutions, Chatsworth, CA), according to Bertoz et al. [29]. CT scans had improved diagnosis ability and the capacity to measure airway volume and precisely observe upper airway structures [30]. Three-dimensional morphological assessments of the maxilla and mandible were carried out using two open-source software by an experienced orthodontist (*ITK-SNAP,* www.itksnap.org and 3D *SLICER,* www.slicer.org*).* Head orientation of the scans in the same Cartesian system was performed as described elsewhere [31].

**Fig. 1:**
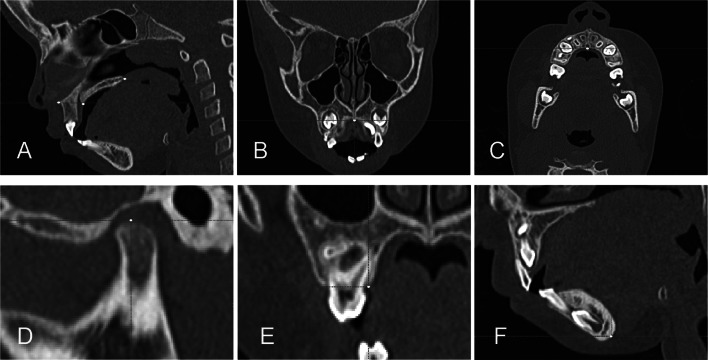
Volumetric anatomical landmarks of the nasal cavity, nasopharynx, and oropharynx volume in axial, coronal, and sagittal views

T1 scan was manually approximated to T0 (best-fit) in the multiplanar views. Then a fully automated voxel-based registration was performed using the anterior cranial base for total superimposition [32] and the body of the mandible for regional superimposition [33]. Four maxillary and two mandibular landmarks were located simultaneously in the sagittal, coronal, and axial views (Fig. 1). The landmarks were as follows:

1. Anterior nasal spine (ANS) (Fig. 1A);
2. Posterior nasal spine (PNS) (Fig. 1A);
3. Palatal point (PP): mid-palatal point below the nasal septum (Fig. 1B e 1C);
4. Condylion (Co) (Fig. 1D);
5. Alveolar point (AL): the most inferior point of the alveolar process of the palatal surface of the second deciduous molar, on the right and left sides (Fig. 1E);
6. Menton (Me) (Fig. 1F).

The assessment of the displacement of the landmarks between T0 and T1 was carried out, taking into account the projected linear displacement of landmarks calculated in the X (right-left), Y (anterior–posterior), and Z (superior-inferior) planes; and also the Euclidean 3D displacements. The angular changes of three constructed planes were estimated as pitch (rotation over the X-axis), roll (rotation over the Y-axis), and yaw (rotation over the Z-axis). The following measurements were assessed: (a) palatal angle: the angle formed by PP and AL; (b) palatal point displacement: displacement of PP; (c) palatal length: Euclidean distance between ANS and PNS; (d) menton–condylion length: distance between Co and Me.

**Fig. 2:**
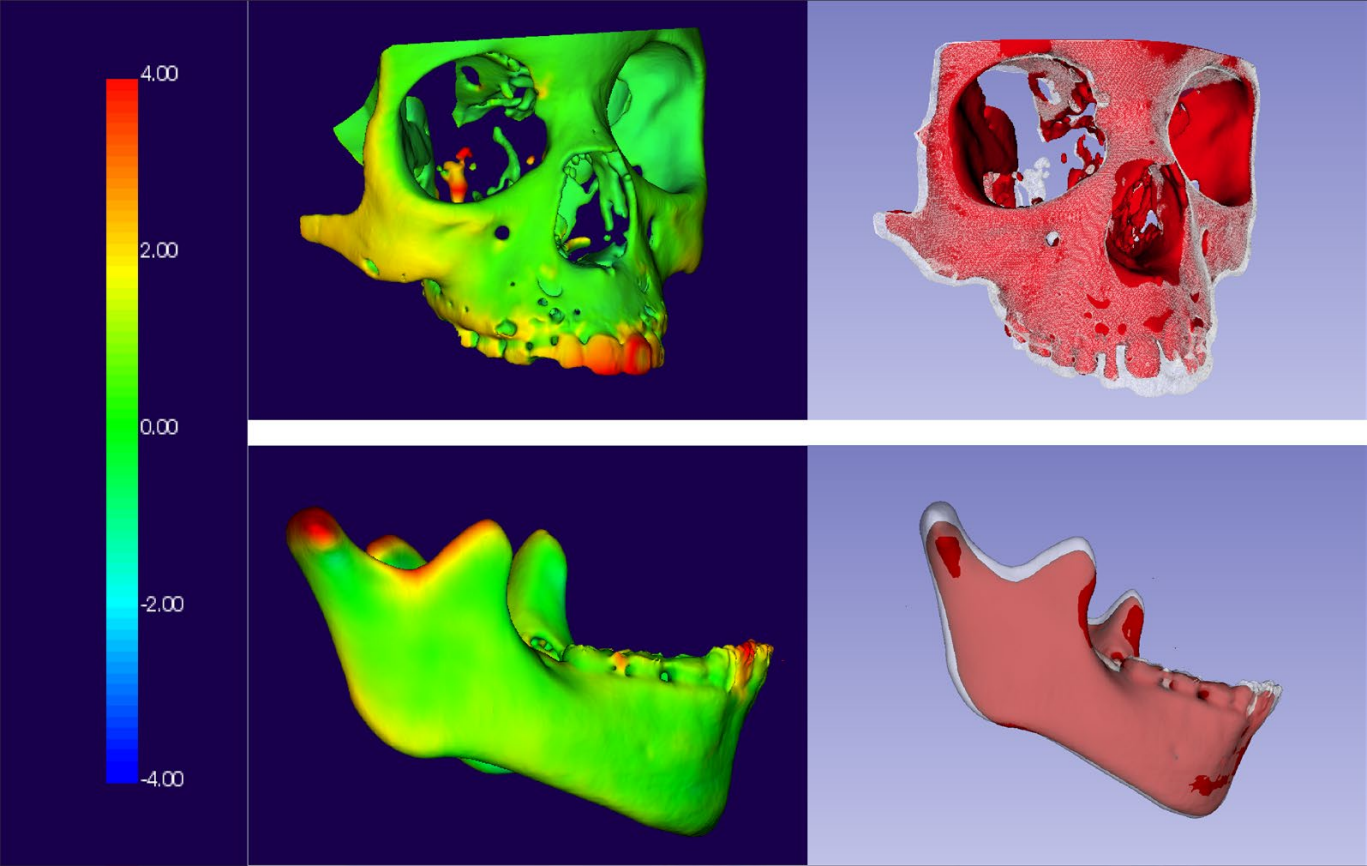
Color maps of virtual models superimpositions

Three-dimensional virtual models were visually analyzed with color maps generated from the superimposition of T0 and T1 models. No displacements between models were indicated with green. Outward displacements from T0 to T1 were shown in hot colors (red/yellow), while inward displacements were shown in cold colors (dark/light blue) (Fig. 2).

## Statistical analysis

Data analysis was performed using SPSS (version 20.0; SPSS, Chicago, Ill). At least one month after completing the first analysis of all cases, the same investigator repeated the 3D image analysis of 10 randomly selected patients to calculate the method’s repeat using an intraclass correlation coefficient (ICC) with a confidence level of 95%. The paired *t*-test was used to test systematic errors, while the method of moments (MME) was used to test the random error. The Kolmogorov–Smirnov test did not confirm the normal distribution of the variables. The Wilcoxon test was used to evaluate the changes in variables over time between T0 and T1. The significance level was set at 5% (*P* < 0.05).

## Results

The ICC showed agreement between readings (greater than 0.80) in the X-, Y-, and Z-axes. Random errors ranged between 0.04 mm (point Co in the Z-axis) and 0.34 mm (point PP on the Y-axis). There were no statistically significant systematic errors between the two measurements performed by the same operator (*P* > 0.05).

Changes in the pulmonary artery systolic pressure, nasal inspiratory flow, upper airway volume, and obstructive apnea/hypopnea index between T0 and T1 are shown in Table 1. Significant changes from T0 to T1 were found. The PSAP was reduced 16.6% in T&A patients and 7.9% in non-T&A patients. NIF increased in both T&A children (40.3%) and non-T&A children (16.8%), respectively, but the highest increase was in the T&A individuals. The obstructive apnea/hypopnea index did not decrease in six T&A children (55%) and three non-T&A children (75%). The mean minimum saturation (%SpO2) before surgery (T0) was 87.5 and after tonsillectomy it measured 90.2 in surgical patients, and 86.6 in non-surgical patients.

**Table 1:** Changes in PSAP, NIF and OAHI between T0 and T1

Variables	PASP			NIF			OAHI		
	TO	T1	% decreased	TO	T1	% increased	T0	T1	T1–T0
T&A									
1	27.0	19.8	26.6%	96.0	246.0	60.9%	1.3	1.1	0.2
2	24.0	21.0	12.5%	179.0	245.0	26.9%	5.5	3.8	2.8
3	26.0	22.0	15.3%	222.0	389.0	42.9%	3.2	2.2	1.0
4	25.0	19.0	24.0%	198.0	259.0	23.5%	2.5	1.5	1.0
5	25.0	22.0	12.0%	153.0	232.0	34.0%	1.2	0.8	0.4
6	26.0	21.0	19.2%	90.0	165.0	45.5%	1.4	0.2	1.2
7	25.0	22.0	12.0%	130.0	272.0	52.2%	1.9	1.8	0.1
8	27.0	24.0	11.1%	106.0	199.0	46.7%	2.5	2.8	0.0
9	29.0	21.0	27.5%	144.0	201.0	28.3%	0.8	0.8	0.0
10	27.0	25.0	7.4%	238.0	381.0	37.5%	1.1	1.0	0.1
11	26.7	22.4	16.1%	121.0	221.0	45.2%	5.8	2.9	2.9
**Mean**	**26.1**	**21.7**	**16.7%**	**152.4**	**255.4**	**40.3%**	**2.4**	**1.7**	**0.7**
Non T&A									
12	30.0	28.0	6.6%	278.0	328.0	24.0%	3.9	1.2	2.7
13	21.0	23.3	9.8%	185.0	220.0	15.9%	1.0	1.0	0.0
14	26.0	23.0	11.5%	104.0	137.0	15.2%	2.2	2.2	0.0
15	27.0	26.0	3.7%	248.0	283.0	12.3%	1.2	0.9	0.3
**Mean**	**26.0**	**25.0**	**7.9%**	**203.7**	**242.0**	**16.8%**	2.0	1.4	**0.6**
*P* value T0-T1	***P* < .001**			***P* < .001**			***P* < .01**		

Mean results are shown in bold

T&A = adenotonsillectomy, T0 = surgical indication, T1 = 18 months later, PASP = pulmonary artery systolic pressure (mmHg), NIF = nasal inspiratory flow (cm.^3^_/s_), OAHI = obstructive apnea/hypopnea index (event/hour)

Table 2 shows the changes in the nasal cavity (NC), nasopharynx (NP), and oropharynx (OP) between T0 and T1. The increase in the dimensions of the airways from T0 to T1 was statistically significant. The volumetric measurements of the upper airway volume increased over time in the NC (30.7%), NP (45.6%), and OP (25.5%) in T&A patients. In non-T&A patients, the volumetric measurements also increased in the NC (10.5%), NP (25.1%), and OP (17.7%).

**Table 2:** Changes in NC, NP and OP between T0 and T1

Variables	NC			NP			OP		
	TO	T1	% increased	TO	T1	% increased	TO	T1	% increased
T&A									
1	12,179.2	13,647.2	10.7%	2811.0	4840.9	41.9%	4736.2	6563.0	27.8%
2	9930.9	11,865.5	16.3%	3230.3	4949.1	34.7%	4496.0	6767.2	33.5%
3	12,101.0	13,557.1	10.7%	3197.5	5008.4	36.1%	5214.2	6450.8	19.1%
4	4182.3	9606.4	56.4%	1855.2	3875.1	52.1%	4390.9	6378.5	31.1%
5	8442.1	12,574.3	32.8%	1910.1	4298.2	55.5%	5142.5	5832.5	11.8%
6	10,078.9	12,366.4	18.5%	3341.1	8854.9	62.2%	3752.4	6366.4	41.0%
7	14,582.0	23,127.5	36.9%	3906.1	7391.7	47.1%	3397.5	4707.7	27.8%
8	11,376.9	21,203.0	46.3%	1929.7	4244.4	54.5%	2745.6	3477.9	21.0%
9	8328.5	13,557.1	38.5%	3070.7	9046.5	66.0%	5613.6	7887.6	28.8%
10	10,626.1	12,466.2	14.7%	3665.5	4920.2	24.5%	8809.0	10,097.0	12.7%
11	7946.5	11,100.7	28.4%	1735.9	2352.4	26.2%	5839.3	7946.5	26.5%
**Mean**	**9979.4**	**14,097.4**	**28.2%**	**2786.6**	**5434.7**	**45.6**%	**4921.5**	**6588.6**	**25.5**%
Non T&A									
12	14,622.7	15,886.7	7.9%	2955.3	4839.,8	38.9%	7215.3	8707.0	17.1%
13	9376.1	9489.1	1.1%	2420.9	3037.4	20.2%	5046.7	5998.8	15.8%
14	10,162.4	11,534.2	11.8%	2157.0	3160.9	31.7%	4620.5	5496.1	15.9%
15	8614.7	10,938.1	21.2%	3100.8	4631.7	33.0%	5398.0	6941.2	22.2%
**Mean**	**10,693.9**	**11,962.0**	**10.5**%	2658.5	**3917.4**	**30.9%**	**5570.1**	**6785.7**	**17.7**%
*P* value T0-T1	***P* < .001**			***P* < .001**			***P* < .001**		

Mean results are shown in bold

T&A = adenotonsillectomy, T0 = surgical indication, T1 = after 18 months, *NC* Nasal cavity (mm^3^), *NP* Nasopharyngeal cavity (mm^3^), *OP* Oropharyngeal cavity (mm^3^)

**Fig. 3:**
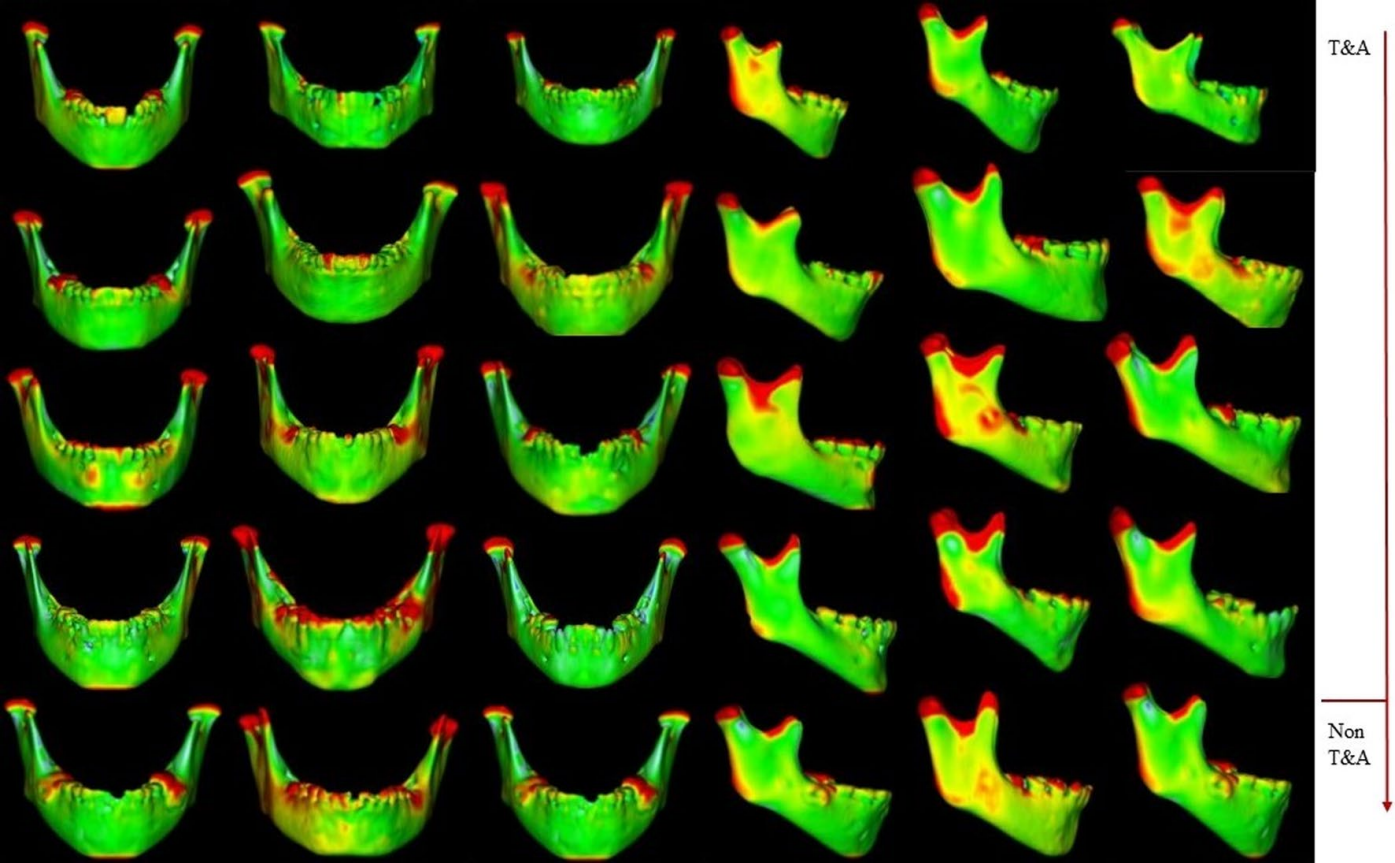
Color map of OSA case series

Three-dimensional measurements of maxillary and mandibular landmarks between T0 and T1 virtual models are presented in Table 3. Palatal angle did not change from T0 to T1, while the other skeletal parameters presented significant modifications over time. The palatal angle increased in T1 in six T&A children (55%) and two untreated children (50%). Downward movement and 3D displacement of the palatal point in these patients were observed. Palatal length and menton–condylion length were slightly greater in T1 compared to T0. The palatal length increased 2.3 mm in T&A children and 2.6 mm in non-T&A children, respectively. The menton–condylion length increased 5.4 mm in T&A patients and 5.5 mm in non-T&A patients. The Co point showed displacement in T&A and non-T&A individuals between T0 and T1. Color maps showed similar changes in T&A and non-T&A patients (Fig. 3).

**Table 3:** 3D displacement of maxillary and mandibular landmarks between T0 and T1

Variables	Palatal Angle			Menton-Condyle length			Palatal point	Condylion point	ANS-PNS
	TO	T1	Increased	T0	T1	Increased	Displacement	Displacement	Displacement
T&A									
1	113.7	130.3	**16.6**	92.8	99.5	**6.7**	**7.0**	**6.2**	0.8
2	104.9	123.9	**19.0**	103.3	105.9	2.6	**6.1**	2.9	2.4
3	125.8	125.1	-0.7	95.5	102.1	**6.6**	2.3	**6.3**	2.4
4	116.8	120.2	3.4	93.9	99.4	**5.5**	3.0	**6.2**	2.7
5	122.1	122.1	0.0	96.5	102.2	**5.7**	3.8	**6.6**	2.9
6	114.7	115.6	0.9	91.3	97.4	**6.1**	4.0	**6.4**	2.9
7	96.6	118.1	**21.5**	98.7	105.1	**6.4**	**8.4**	**6.6**	3.2
8	110.6	133.3	**22.7**	88.5	95.0	**6.7**	**9.4**	**6.4**	2.9
9	114.5	115.1	0.6	102.3	102.7	0.4	0.1	2.4	0.1
10	104.6	124	**19.4**	104.9	111.8	**6.9**	**8.0**	**6.8**	2.6
11	58.5	66.4	**7.9**	92.1	98.3	**6.2**	**10.2**	**7.1**	3.0
**Mean**	**107.5**	**117.6**	**10.4**	96.3	**101.7**	**5.4**	**5.6**	**5.8**	**2.3**
Non T&A									
12	115.2	133.7	**18.5**	97.0	104.0	**7.0**	**9.6**	**6.6**	1.8
13	52.1	63.2	**11.1**	96.4	96.6	0.2	**5.9**	0.2	4.6
14	105.9	106.7	0.8	89.4	97.0	**7.6**	1.7	**6.4**	1.9
15	118.4	124.7	6.2	90.1	97.6	**7.5**	3.6	**6.3**	2.4
**Mean**	**97.9**	**107**	**9.1**	93.2	**98.8**	**5.5**	**5.2**	**4.8**	**2.6**
*P* value T0-T1	***P* > .05**				***P* < .001**		***P* < .001**	***P* < .001**	***P* < .001**

Mean results are shown in bold and the most representative results of the changes between T0 and T1 in some children were also highlighted

T&A = adenotonsillectomy, T0 = surgical indication, T1 = 18 months later, Palatal angle (°), Mento-Condyle length (mm), Palatal point displacement (mm), Condylion point displacement (mm), ANS-PNS (anterior nasal spine-posterior nasal spine) (mm)

## Discussion

Despite the small sample size, the current study presents unique and relevant information not previously presented regarding echocardiography, polysomnography, rhinomanometry, and computed tomography imaging. This case series report presents a novel view of several clinical parameters and facial morphology after the normalization of the mode of breathing of OSA children over 18 months after surgical intervention.

Changes in the respiratory dynamics during sleep may result in hypoxemia and hypercapnia that leads to vasoconstriction of the pulmonary artery and may progress to pulmonary hypertension. In this study, the PASP decreased in all T&A children, and this reversibility of the PASP value corroborates the literature data. Previous studies investigating children with OSA assessed the progression of pulmonary artery systolic pressure over time, suggesting that PASP levels can be reversed after surgical intervention and may decrease six months after adenotonsillectomy [11–13]. After surgery, the change in cardiovascular parameters indicates signs of early cardiovascular dysfunction in patients with OSA. It is unknown whether increased pulmonary pressure levels in childhood, even within normal limits, may contribute to future cardiovascular morbidity. Nevertheless, it is also possible that as children grow up, their nasal patency increases and adenotonsillar obstruction decreases, which could explain the PASP reduction in non-T&A patients.

This study showed a tendency for increased nasal inspiratory flow over time between surgical patients and non-surgical patients. Increased nasal inspiratory flow may be associated with removing the obstructive factor (surgical patients). The craniofacial growth may also be associated with this increase in nasal flow. The aging of the airflow may also be associated with reducing the lymphoid tissue in non-operated children [11, 12]. This group’s improvement also corroborates that watchful waiting may be rationale in mild cases.

The obstructive apnea/hypopnea index (OAHI) did not decrease in six T&A children and three non-T&A children. Clinical improvements were reported despite the persistence of altered OAHI. The removal of the obstructive factor contributed to the improvement in the mode of breathing, which can be confirmed by the minimum saturation (%SpO2) increase in the T&A children; however, the OAHI reduction was not found. Previous studies have also reported the persistence of OSA in T&A children [10, 11]. In these studies, improvements in symptoms occurred independently of the normalization of OAHI. Possibly the patients who underwent surgery improved due to the removal of the obstructive factor even without normalization of the OAHI. Many factors may impact the index, but not necessarily the improvement in the quality of the breathing following T&A. Impairment of orofacial muscle’s function has also been cited as one of the maintenances and relapse factors of childhood OSA [5].

Volumetric upper airway measurements increased over time in the nasal cavity, nasopharynx, and oropharynx. It may be associated with removing the obstructive factor (adeno/-tonsillectomy) and increasing the airflow through the airways. Nasopharynx volume increased the highest percentage in the T&A children, which may be associated with the surgery (T&A). In non-T&A patients, the upper airway volume increased the volumetric measurements, probably due to craniofacial growth or decreased pharyngeal lymphoid tissue in the older children. Still, the ratio of changes was smaller than in the T&A group. The percentage of increased airways compartments were greater in individuals submitted to T&A.

There were no significant changes in the maxillomandibular growth pattern after the T&A. Predominance of green colors was found in craniofacial color maps of T&A children, meaning small changes after T&A. Qualitative 3D assessment showed the same pattern of facial changes in T&A and non-T&A patients. This result corroborates recent publications that reported no significant differences in dentofacial morphology after the surgical normalization of the mode of breathing [16–19].

Minor displacements between landmarks from T0 and T1 suggested that no significant skeletal changes in dentofacial structures occur after adenotonsillectomy. In our study, the palatal angle has become more obtuse in 55% of T&A children and 50% of untreated children in T1, resulting in a less constricted maxillary and a more open palate. As a result, 3D vertical displacement of PP was seen to be associated with the vertical growth of the maxillary. In all children, the menton–condylion length increased over time, and the Co points showed significant spatial displacement due to excessive growth in this area.

The small number of subjects is the major limitation of the present study. However, the originality of the description, including simultaneously Doppler echocardiography, polysomnography, rhinomanometry, and CT scans of the same mouth breathing patients, is unique. The difficulty of retrieving a sample with the parameters reported in the current investigation is tremendous, making it a pioneer report. The data of untreated mouth breathing patients, which only can be done in retrospective study designs, are also very rare. The goal was not to make statistical inferences about comparing surgical and non-surgical control individuals with a very small sample. Still, the descriptive data were given for a preliminary understanding of the trend of the changes with the normalization of the mode of breathing.

The current finding suggests the need for more data on this topic, with a larger sample in a randomized clinical trial. However, this case series report shows that clinicians should not expect miraculous maxillomandibular growth modifications after T&A. It is important to highlight that hereditary is the major regulator of dentofacial growth. Such assumptions have already been in recent articles by Souki et al. [16]; Tidestrom e Hultcrantz [17]; Mattar et al. [18] and Franco et al. [19].

The current investigation analyzed records collected at an 18-month interval. It can be inferred that such a follow-up period was not enough to produce changes in skeletal growth, but it is necessary to consider that lymphoid tissue decreases with age. Thus, evaluations after several years would carry changes due to normal growth, regardless of surgical approach, without the negative influence of hypertrophic tissue. These findings must be taken with caution due to the small sample and the absence of inferential statistical analysis and more studies are needed to clarify this topic.

The orthodontist needs to understand the factors involved with children’s OSA to provide the appropriate treatment recommendation and judge to what extent the patients can benefit from the interventions. This study suggests that the adenotonsillectomy could improve some clinical parameters, such as pulmonary artery systolic pressure and nasal inspiratory flow, which support the benefits of the surgery in most cases of OSA. However, contrary to what was previously thought, it is often not enough to achieve complete resolution of obstructive apnea/hypopnea index and maxillary and mandibular growth problems.

## Conclusion

Pulmonary artery systolic pressure decreased, nasal inspiratory flow increased, and nasopharynx volume increased following adenotonsillectomy of mouth breathing children. Still, obstructive apnea/hypopnea index and maxillomandibular morphology were similar in surgical and non-surgical patients.

## Availability of supporting data

All data generated or analyzed during this study are included in this article. Further enquiries can be directed to the corresponding author.
